# An Innovative Model of Dementia Programming for Community-Dwelling Older Adults With Family Caregivers

**DOI:** 10.1093/geroni/igaa057.872

**Published:** 2020-12-16

**Authors:** Gregg Gorzelle, Michael Skrajner, Cassie Best, Drew Walker

**Affiliations:** 1 Hearthstone Alzheimer Care, Hiram, Ohio, United States; 2 Hearthstone Alzheimer Care, Wickliffe, Ohio, United States; 3 Hearthstone, Cleveland, Ohio, United States; 4 Hearthstone, Woburn, Massachusetts, United States

## Abstract

Decide, Discover, and Do!TM (D3) is an alpha-version iPad application developed and evaluated in a National Institute on Aging-funded Phase 1 SBIR project. The goal of D3 is to enhance the quality of life and care for community-dwelling persons living with dementia whose primary care partners are family members. D3 consists of (1) evidence-based activities for care partners to facilitate with their loved ones and (2) video-based interactive training on best practices in dementia care, for care partners. The activities are unique in that they create an overarching narrative for daily activities that creates a consistent routine capitalizing on procedural memory. The activities build upon one another, starting with the persons living with dementia choosing a topic (e.g., nature) early in the day, followed by the dyad engaging in a tablet-based activity related to the topic (e.g., reading an article about forests), and culminating in an experiential activity (e.g., tasting various foods found in nature, e.g. wild raspberries). A total of 18 participants took part in the this feasibility study. The study examined the impact of D3 training modules on knowledge transfer (16% mean increase in care partner knowledge across three courses); satisfaction with the training modules (96% satisfaction across three courses); and satisfaction with the activities programming (91% satisfaction across all items for persons living with dementia and 99% for care partners). No distal changes related to care partner stigma nor caregiver burden were seen in this short study.

